# Recurrent *Pasteurella multocida* urinary tract infection in a young adult with end-stage renal insufficiency: a case report

**DOI:** 10.1128/asmcr.00217-25

**Published:** 2026-02-26

**Authors:** Shaindel Minkowski, Gaëlle Cuzon, Thomas Droit, Lélia Escaut, Agnès B. Jousset

**Affiliations:** 1 Service de Maladies Infectieuses et Tropicales, Assistance Publique-Hôpitaux de Paris, Hôpital Bicêtre, Université Paris-Saclay 27048, Le Kremlin-Bicêtre, France; 2 Service de Bactériologie-Hygiène, Assistance Publique-Hôpitaux de Paris, Hôpital Bicêtre, Université Paris-Saclay 27048, Le Kremlin-Bicêtre, France; 3 Université Paris-Saclay, Inserm, CEA, Center for Immunology of Viral, Auto-immune, Hematological and Bacterial diseases (IMVA-HB/IDMIT/UMRS1184) 27048, Fontenay-aux-Roses & Le Kremlin-Bicêtre, France; Rush University Medical Center, Chicago, Illinois, USA

**Keywords:** *Pasteurella multocida*, urinary tract infection, cat exposure, whole-genome sequencing, case report

## Abstract

**Background:**

*Pasteurella multocida* is a gram-negative coccobacillus that colonizes the oropharyngeal and digestive tract of animals, particularly cats and dogs. It is commonly associated with mucocutaneous and osteoarticular infections.

**Case Summary:**

We report the case of a 25-year-old man with a history of obstructive uropathy and end-stage renal disease awaiting transplantation, who presented to the emergency department twice, 8 months apart, with afebrile back pain. On each admission, urine cultures grew *Pasteurella multocida*. Whole-genome sequencing of both isolates revealed only two single-nucleotide polymorphisms, supporting a recurrence caused by the same strain. He was treated with injections of ceftriaxone followed by fluoroquinolones for a total of 14 days. Further history revealed frequent cat bites and scratches in the household. Previously published cases of urinary tract infections due to *Pasteurella* sp. involved most often patients with underlying urological abnormalities. Contact with a pet was not consistently reported.

**Conclusion:**

This case underscores that *P. multocida* can cause recurrent urinary tract infection in immunocompromised patients with pet contact.

## INTRODUCTION

*Pasteurella* spp. are gram-negative coccobacilli that colonize the mucosal surfaces of animals, particularly cats and dogs, but also other mammals such as felines, rats, opossums, rabbits, and horses (1). There are four main species involved in human infections: *Pasteurella multocida*, *Pasteurella canis*, *Pasteurella dagmatis*, and *Pasteurella stomatis*. Human infection usually follows a bite, scratch, or direct contact with animal secretions, and the majority of cases present as localized skin soft tissue infections or, less frequently, as respiratory disease (1).

Determining the prevalence of *Pasteurella* spp. infections and *Pasteurella multocida* is challenging due to underreporting of minor wounds, especially those related to pets, which are not systematically microbiologically documented. Nevertheless, the number of *Pasteurella* spp. infections appears to be increasing worldwide (1). Urinary tract infection (UTI) caused by *Pasteurella* is exceptionally rare. An observational study conducted in France over an 8-year period (1985 and 1992) described 1,153 Pasteurella spp. infections, of which two-thirds were local inoculation forms and one-third systemic infections. Among the 1,191 strains included, only 6 were isolated from urine samples, confirming the rarity of this type of infection (2).

We therefore present a case of recurrent *P. multocida* UTI in a young man with end-stage renal disease awaiting transplantation, confirmed by genomic analysis, and we provide a systematic review of all reported *Pasteurella* urinary infections to delineate their clinical spectrum and risk factors.

## CASE PRESENTATION

A 25-year-old male patient, with a history of congenital obstructive uropathy (posterior urethral valves) corrected surgically in infancy, was followed for chronic kidney disease. He underwent a Mitrofanoff continent catheterization (first performed in 2009, later revised in 2021 because of stenoses) and a left radiocephalic arteriovenous fistula for hemodialysis that started in early 2023. He was awaiting a pre-transplant workup at the time of presentation.

The patient came to the emergency department with afebrile back pain radiating to the left flank. Urine cytobacteriological examination revealed leukocyturia >10^6^/mL and numerous gram-negative bacilli. The following day, small round, smooth colonies grew on chromogenic medium (CPSO, Biomérieux). Identification by matrix-assisted laser desorption/ionization time-of-flight mass spectrometry (Microflex LT mass spectrometer, Bruker Daltonics, Wissembourg, France) yielded *Pasteurella multocida* with a high-confidence score >2. Antibiotic susceptibility testing (AST) by disk diffusion on Mueller-Hinton agar supplemented with horse blood (Bio-Rad) demonstrated susceptibility to all tested antibiotics, including amoxicillin, cefotaxime, ciprofloxacin, tetracycline, and cotrimoxazole. Clinical categorizations were interpreted using EUCAST v2025 guidelines.

The patient was discharged after two intramuscular injections of ceftriaxone administered in the emergency department and a prescription of ceftriaxone 1 g/day for 48 h. Once AST results became available within 48 h, his general practitioner prescribed fluoroquinolones to complete a 14-day course. The patient did not return for follow-up, as his symptoms resolved without recurrence.

**Fig 1:**
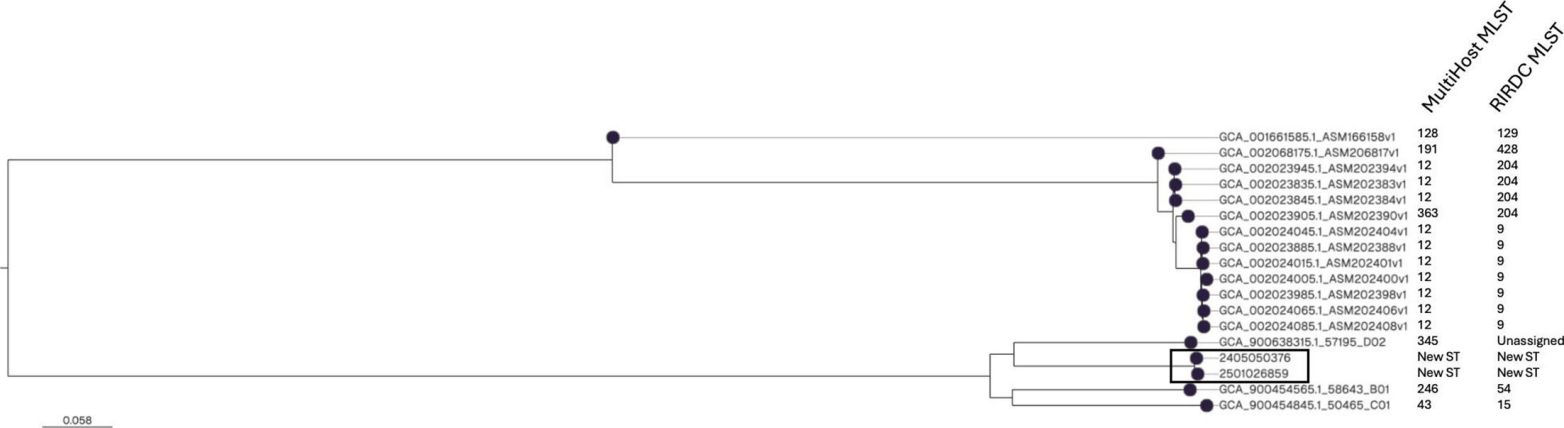
Phylogenetic analysis of the two *Pasteurella multocida* subsp. *septica* isolates recovered from urine samples of the same patient 8 months apart (2405050376 and 2501026859, circled), along with all available *P. multocida* subsp. *septica* genomes from the NCBI database. Sequence types (STs) are indicated according to both the RIRDC and Multiple Host MLST schemes.

Interestingly, retrospective analysis of the patient’s medical records revealed a similar episode 8 months earlier, with urine culture also positive for *P. multocida* and an identical susceptibility profile. To determine whether this represented reinfection or recurrence with the same strain, whole-genome sequencing was performed using Illumina technology. Genomic analysis identified *P. multocida* subspecies *septica*. Single-nucleotide polymorphism (SNP) analysis using CLC Genomics Workbench revealed only two SNPs between the two isolates, supporting a clonal relationship (Fig. 1). Comparative genomic analysis was performed using publicly available genomes of *P. multocida* subsp. *septica* (*n* = 16) with CSI Phylogeny (https://cge.food.dtu.dk/services/CSIPhylogeny/), confirming the close genetic relationship between the two patient isolates (Fig. 1). Multilocus sequence typing (MLST) analysis, performed using both schemes available at http://pubmlst.org/pmultocida/, showed that the isolates did not correspond to any known ST. The closest matches were ST-25 (5/7 loci ; 71.4% identity) according to the RIRDC MLST scheme and ST-279 (3/7 loci ; 42.9% identity) with the Multiple host MLST scheme.

On further questioning, the patient reported long-term ownership of a domestic cat that frequently scratched and bit him, although no direct contact with the Mitrofanoff catheter or its cutaneous exit site was reported.

## DISCUSSION

*P. multocida* is a facultative anaerobe gram-negative coccobacillus, commonly found as a commensal in the nasopharyngeal tract of domestic pets, particularly cats and dogs. Transmission can occur through direct inoculation via bites or scratches, but also through contact with contaminated saliva, even in the absence of skin breach (1).

Although dog bites are more frequent, infection occurs in only 3%–18% of cases, whereas cat bites become infected in 20%–80% of cases (3, 4), probably due to the anatomy of feline claws that allow for deeper inoculation, and the higher colonization rates observed in cats. In fact, *P. multocida* is the most frequently isolated microorganism in infections following cat bites, found in 50%–80% of cases (5). The colonization rate of *P. multocida* is higher in cats than in dogs (70%–90% vs. 20%–50%) (6).

Twenty cases of *Pasteurella* spp. urinary tract infection were reported between 1967 and 2022 (no other case of *Pasteurella* spp. UTI was reported after 2022); these cases are presented in Table 1. These comprise 18 adults and two pediatric patients (aged 13 and 15 years), with a female predominance (13 women vs. 7 men). These infections appear to be favored by underlying urological conditions: a history of lower urinary tract malignancy (cervical, bladder, and prostate cancer) was reported in 7/20 cases; neurological disorders, potentially associated with neurogenic bladder or dysuria in 4/20 cases; diabetes mellitus in 3/20 cases; and obstructive uropathy with chronic kidney disease in the two pediatric cases and our patient. The majority of infections (19/20) were caused by *P. multocida*, confirming its predominant role among *Pasteurella* spp. Of the 15 cases with available exposure history, only one involved a documented dog bite. In the remaining cases, no recent scratch or bite from a cat or dog was reported, even among the 9/20 other pet owners.

**TABLE 1:** *Pasteurella* spp. urinary tract infections

Reference	No. of cases (*n* = 20)	Age, gender	Medical condition	Causative microorganism	Animal exposure	Treatment
7	1	62, female	Uterine cervical carcinoma with non-functional kidney, radiation therapy, bladder metastasis, hydronephrotic left kidney	*P. multocida*	Cat (no recent history of bites or scratches)	1.5 g of ampicillin IV for the first 24 h, then 500 mg ampicillin/8h, PO—10 days
8	5	Female no. 1 Female no. 2 Female no. 3 Female no. 4 Male no. 5 (age unknown for all)	1. Pneumonia 2. Dysuria 3. Chronic cystitis 4. Carcinoma of the bladder with nephritis 5. Paraplegia, chronic pyelonephritis	All *P. multocida*	Unknown for all	Unknown for all
9	3	63, male no.1 71, male no. 2 51, male no. 3	1. Rheumatic valvular heart disease 2. Insulin-dependent diabetes, indwelling Foley catheter following transurethral prostatic resection for hypertrophy 3. Insulin-dependent diabetes, recurrent peptic ulcer	*P. multocida* *P. haemolytica* *P. multocida*	No recent animal contact No recent animal contact No recent animal contact	600,000 units of penicillin intramuscularly twice a day for 7 days Intravenous gentamicin and oxacillin 600,000 units of penicillin intramuscularly twice a day; unknown duration
10	1	15, male	Obstructive uropathy with chronic renal failure requiring hemodialysis, left kidney removed at 6 months, ileal loop cutaneous urinary diversion, recurrent UTIs (urinary tract infections)	*P. multocida*	1 cat, 1 kitten, and 1 rabbit; throat culture from the cat was positive for *P. multocida*, negative for the other two animals	70 mg gentamicin IM once
11	1	77, female	Hypertension	*P. multocida*	Unknown	Trimethoprim-sulfamethoxazole, 10 days
12	1	26, female	Uterine cervical carcinoma, pelvic irradiation complicated by ileo-vaginal fistula, repaired by ileal bypass	*P. multocida*	Dogs and cats, uninfected dog bite 1 year prior	Trimethoprim/sulfamethoxazole, unknown duration
13	1	31, female	Smoker 20-pack-year, left pyelonephritis 10 years earlier	*P. multocida*	Dog (slept in her bed and occasionally drank water from the commode)	Amikacin 500 mg IV/12 h, cephalothin 1 g/6 h for 24 h, cephradine 500 mg/6 h PO for 14 days total
14	1	25, female	Acute leukemia, brain and pelvic metastasis, hysterectomy and bilateral cutaneous ureterostomy, Kock pouch (continent ileostomy), recurrent UTIs	*P. multocida*	None	Amoxicillin 3 g/day and pefloxacine 1.2 g/day; unknown duration
15	1	56, female	Uterine cervical cancer with pelvic exenteration, Indiana pouch with urinary diversion	*P. multocida*	2 cats (no recent history of bites or scratches); proved mouth colonization by *P. multocida*, with high degree relationship (100% band match)	Unknown
3	1	13, male	Obstructive uropathy (posterior urethral valves) with chronic renal failure, peritoneal dialysis, nephrectomy of the left dysplastic kidney at 3 years of age, Mitrofanoff procedure at 4, recurrent UTIs	*P. multocida*	Cat (no recent history of bites or scratches)	Amoxicillin–clavulanate, 14 days
4	1	59, female	Uterine cervical cancer status post colostomy and urostomy with Kock pouch (continent ileostomy), nephrolithiasis, and UTIs	*P. multocida*	6 cats (no recent history of bites or scratches)	Oral amoxicillin 500 mg/8 h, 10 days
16	1	48, female	Uterine cervical cancer	*P. multocida*	Cats (no recent history of bites or scratches)	Trimethoprim-sulfamethoxazole (800/160 mg), 14 days
17	1	83, male	Prostatic adenoma and inguinal hernia, recurrent UTIs	*P. multocida*	Dog (no recent history of bites or scratches); proved mouth colonization by *P. multocida* (same strain proved)	Oral ciprofloxacin, 500 mg every 12 h for 7 days
18	1	34, female	Non-insulin dependent diabetes mellitus, spina bifida with hydrocephalus, congenital paraplegia, neurogenic bladder, horseshoe kidney, renal calculi, recurrent UTIs	*Klebsiella oxytoca, P. multocida*	Cat (no recent history of bites or scratches), skin licking	Amoxicillin-clavulanic acid, 7 days

*Pasteurella* is susceptible to penicillin, amoxicillin, cephalosporins (except cephalexin), tetracycline, cotrimoxazole, and chloramphenicol. Macrolides (clindamycin and erythromycin) show poor activity. Among aminoglycosides, gentamicin is the most active. Regarding acquired resistance, a β-lactamase can be found but remains rare (19). For soft tissue infections, which are often polymicrobial, broad-spectrum antibiotics are recommended. These include amoxicillin-clavulanic acid, or in case of penicillin allergy, doxycycline plus metronidazole, or clindamycin combined with a fluoroquinolone (ciprofloxacin), with alternatives such as trimethoprim-sulfamethoxazole for children or ceftriaxone for pregnant women (1, 20).

There are currently no specific treatment guidelines for *Pasteurella* urinary tract infections. However, based on available susceptibility data, the use of β-lactams, fluoroquinolones, or cotrimoxazole appears appropriate due to their diffusion in the urine (1).

### Conclusions

This report of a UTI caused by *Pasteurella* spp. emphasizes three clinically relevant points. First, a previous infection does not preclude recurrence, even with the same strain. Second, *Pasteurella* infections can occur in the absence of bites or scratches. Simple contact with a colonized pet may be sufficient, particularly in patients with congenital uropathy, pelvic malignancy, or urological devices. In clinical practice, hygiene precautions should be reinforced in pet owners with such risk factors. Third, despite the absence of formal guidelines, treatment remains straightforward given the rarity of acquired resistance. Amoxicillin, amoxicillin-clavulanate, cotrimoxazole, and fluoroquinolones are effective options, typically administered for 7–14 days, with clinical reassessment. Several questions remain unanswered, including the potential role of vaccination in at-risk individuals with frequent cat exposure, given that vaccines are already in use in veterinary medicine (21).

## Data Availability

Genomes of both isolates were deposited in the NCBI database under BioProject no. PRJNA1292213.
